# Spectrophotometric Methods for the Determination of Cefprozil in Bulk and Dosage Form

**Published:** 2009-09

**Authors:** Elrasheed A. Gadkariem, Mohammed M. Mutasim, Kamal E. E. Ibrahim, Humeida A. El-Obeid

**Affiliations:** 1 *Department of Pharmaceutical Chemistry, Faculty of Pharmacy, University of Khartoum, Sudan;*; 2 *Department of Pharmaceutical Chemistry, University of Medical Sciences and Technology, Sudan*

**Keywords:** determination, spectrophotometry, liquid chromatography, ascorbic acid, sodium hydroxide, kinetics, cefprozil, tablets

## Abstract

Two simple spectrophotometric methods were developed for the determination of cefprozil in pure bulk and in tablets forms. The first is a colorimetric method based on the coupling of cefprozil, after being hydrolyzed by sodium hydroxide (0.1N), with ascorbic acid as a chromogen (method A). It has been established that cefprozil reacts with ascorbic acid to form a 1:1 water soluble colored product with maximum absorbance (λ_max_) at 408 nm and molar absorptivity of 7.2 × 10^3^L mol^−1^ cm^−1^. The second method (Method B) utilizes a direct reaction between cefprozil and sodium hydroxide (1N). A colored product with λ_max_ at 486 nm and molar absorptivity of 7.4 × 10^3^ L mol^−1^ cm^−1^ is formed after heating cefprozil with sodium hydroxide (1N). The absorbance-concentration plot was rectilinear over the range 5–25 µg/ml in both methods with correlation coefficient values not less than 0.999. The detection limits (L.O.D.) were 0.96 µg/ml and 0.93 µg/ml for method A and method B respectively. The methods were validated using the USP liquid chromatography method for cefprozil assay. The results obtained by the USP liquid chromatography method for the tablets dosage form were statistically compared with those of the developed methods and evaluated at 95% confidence limits.

## INTRODUCTION

Cefprozil is a semi-synthetic broad-spectrum, second generation cephalosporin antibiotic (1). It is used as the cis- and trans-isomeric mixture (≤90% cis). The chemical name for the monohydrate is (6R, 7R)-7-[(R)-2-amino-2-(p-hydroxyphenyl)acetamido]-8-oxo-3-propenyl-5-thia-1-azabicyclo[4-2-0]oct-2-ene-2-carboxylic acid monohydrate.

Cefprzil is an orally active cephalosporin which is used in clinical practice. It belongs to the β-lactam group of antibiotics. Its antibacterial activity is dependent on the presence of the β-lactam functionality which is not stable in aqueous conditions. This instability leads to chemical degradation and the formation of 2,5 – dione derivative via intra molecular nucleophilic attack of the primary amine from the side chain of the lactam moiety at neutral or slightly alkaline medium (2–3).

Numerous analytical procedures have been reported for determination of cephalosporin in pure form, pharmaceutical formulations and biological fluids (4). The methods reported for the quantitative determination of cefprozil in pharmaceutical formulations and biological fluids include chromatography (5–7) spectrophotometry (8–10) and a chemiluminescence method using flow injection analysis (11).

Ascorbic acid has been frequently utilized as an analytical reagent in pharmaceutical analysis. Reaction of ascorbic acid with penicillins and cephalosporins having α-aminoacyl functions have been reported under both acid and alkaline media (12, 13). Based on these reported results, simple, sensitive and selective assay procedures were developed for the determination of the cephalosporin, cefprozil in bulk and tablets formulation.

## EXPERIMENTAL PROCEDURES

### Materials

- Reference cefprozil (99.89% w/w, USP);
- Cefprozil-containing formulation. Cefzil® tablets containing 500 mg cefprozil;
- Dimethylformamide (DMF) was obtained from Merck KGA (Germany);
- Ascorbic acid (BDH), Poole, England. Freshly prepared (2% w/v in DMF);
- Acetonitrile, Hipersolv^(TM)^, BDH, Poole, England;
- Sodium hydroxide (BDH), Poole, England;
- Hydrochloric acid, analytical grade, (BDH) Poole, England;
- Glacial acetic acid, analytical grade, (BDH) Poole, England.

### Instruments

- Jasco V-530 UV/VIS spectrophotometer, Japan;
- Balance, Kern ALS 120-4, Germany;
- Waters 600 liquid chromatograph with Waters 2996 Photodiodarray detector and Enpower integrator. Wavelength 280 nm. A fixed loop injector (Rheodyne, 20 µl) was used to transfer the sample into the column (Varian, C18, 4.6 mm × 25 cm, 5 µm). Mobile phase: Acetonitrile, phosphate buffer pH 4.4 (20:180). Flow rate 1.0 ml/minute;
- Bandelin Sondrex ultrasonic bath, Germany.

### Procedures

**Standard stock solutions:**

- **Method A:** A stock solution of cefprozil (0.05% w/v) was prepared in distilled water. Ten ml of this solution was transferred into stoppered glass tube, 3 ml of 0.1N NaOH were added and the solution was heated in a boiling water bath for 10 minutes; cooled and acidified with 6 ml 0.1N HCl; this solution was transferred quantitatively into a 100 ml volumetric flask and diluted to the mark with distilled water (50 µg/ml, solution a).
- **Method B:** A stock solution of cefprozil (0.05% w/v) was prepared in distilled water. Ten ml of this solution was diluted to 100 ml with distilled water (50 µg/ml, solution b).

**Calibration graphs:**

- **Method A:** Different accurately measured volumes (1–5 ml) of solution (a) were transferred into five stoppered glass tubes. Distilled water was added to make a volume of 5 ml in each tube. Fresh ascorbic acid reagent (1 ml) was then added to each tube before heating at 100°C for 20 minutes using an adjustable water bath. After cooling, the solutions were transferred quantitatively into 10 ml volumetric flasks and the volume was completed to 10 ml with distilled water, mixed well before reading at 408 nm against the appropriate blank (1 ml of ascorbic acid in DMF diluted to 10 ml with distilled water). A graph was constructed by plotting the absorbance values versus drug concentration in µg/ml or these values were computed to obtain a regression analysis data for the graph.
- **Method B:** Different accurately measured volumes (1–5 ml) of solution (b) were transferred into five stoppered glass tubes. Distilled water was added to make a volume of 5 ml in each tube. Sodium hydroxide (1N) (1 ml) was added to each tube before heating at 100°C for 15 minutes. After cooling, the volumes were transferred quantitatively into 10 ml volumetric flasks and the volume was adjusted to 10 ml with distilled water, mixed well before reading at 486 nm against the appropriate blank (1 ml of 1N NaOH diluted to 10 ml with distilled water). A graph was constructed by plotting the absorbance values versus drug concentrations in µg/ml or these values were computed to obtain a regression analysis data for the graph.

**Procedure for tablets:** Twenty tablets of cefprozil were weighed and powdered. An accurately weighed amount of the powdered tablets equivalent to 0.05 g cefprozil was transferred into a 100 ml volumetric flask and shaken with about 70 ml distilled water for 15 minutes. The volume was then completed to 100 ml with distilled water, mixed and filtered.

For the determination of tablets content, solutions of the sample similar to solution (a) [Standard stock solutions (Method A)] and solution (b) [Standard stock solutions (Method B)] were prepared and labeled solutions (c) and (d) respectively. A 3 ml volume of either solution (c) or solution (d) was then treated as under calibration graphs [Calibration graphs (Method A)] or [Calibration graphs (Method B)]. A graph was also constructed from each of solutions (c) and (d), similar to the reference solutions graph, to compare the slopes of these graphs as another means for tablet content determination.

**Difference spectroscopic method (ΔA):** An aliquot of 10 ml of 0.05% w/v cefprozil was treated as under procedures section [Standard stock solutions (Method A)] and section [Calibration graphs (method A)]. The color, however, here, is read against a blank containing the same volume of cefprozil but replacing the 3 ml of 0.1N NaOH with distilled water to get nonhydrolysed drug and acidifying with 3 ml of 0.1N HCl instead of the 6 ml. The concentration of the sample can either be calculated from direct sample/standard comparison as follows:

$$\frac{\Delta \text{A}\;\text{sample}\times \text{C}\%\;\text{standard}}{\Delta \text{A}\;\text{standard}}=\text{C}\%\;\text{intact}\;\text{drug}$$

or from calibration graph of ΔA's versus concentration of the reference sample in the same range of the calibration graphs of the drug.

The ΔA method is applicable to method A only.

**Effect of heating time on the color intensity and consistency (Method A):** Calibration graph procedure described under section [Calibration graphs (method A)] was repeated varying the heating time with ascorbic acid (5, 10, 15 and 20 minutes). The results obtained were recorded. The effect of heating time was assessed through best correlation coefficient value and maximum color intensity for each set of graphs.

**Effect of different concentrations of sodium hydroxide on cefprozil stability (Method B):** Sodium hydroxide solutions (0.2, 0.5 and 1.0N) were used in this study. The effect of these solutions on cefprozil stability was followed utilising the USP liquid chromatography method. A plot of log area of cefprozil versus time (zero time, and different times after addition of sodium hydroxide) was done for each NaOH concentration. The results of this study were utilised to calculate some kinetic parameters like t½, t_90_ and the reaction rate (k_obs_) and also to optimise the suitable normality of NaOH to be used for method B e.g. normality that can cause fast and maximum degradation of the drug which results in maximum color development.

## RESULTS AND DISCUSSION

The importance of cephalosporins and the continuing introduction of new drugs of this series have prompted many researches to explore methods for their determination. Cefprozil, one of these drugs, is the target in the present work. Two methods for its determination in bulk and tablets dosage form were developed.

### Method A

Cefprozil, like some penicillins and cephalosporins, has an α-aminoacyl function. Based on the reported reaction of ascorbic acid with such drugs (13), the reaction of cefprozil with ascorbic acid under the same conditions was investigated. A yellow colored product absorbing maximally at 408 nm was produced.

**Figure 1:**
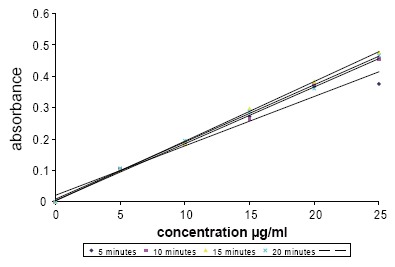
Effect of heating time on calibration curves of cefprozil-ascorbic acid complex.

Factors that can influence the color formation, intensity and stability were studied. These factors included solvent, the reagent concentration, the reaction time and temperature. The suitable sodium hydroxide normality and volume that can quantitatively hydrolyse the drug was also optimized as this is the first step of the reaction with ascorbic acid. After the hydrolysis process, the pH of the solution was acidified with HCl, as ascorbic acid is unstable in alkaline medium. The effect of heating on color production, intensity and the calibration graph best correlation coefficient r-value is presented in Table 1 and Fig. 1.

**Table 1:** Estimation of cefprozil-ascorbic acid complex reaction time and stability (n=2)

Conc. μg/ml	Absorbance/time (minutes)			
	5	10	15	20

5	0.103	0.104	0.103	0.107
10	0.188	0.184	0.186	0.195
15	0.272	0.265	0.297	0.288
20	0.367	0.374	0.383	0.361
25	0.376	0.455	0.476	0.467
Correlation coefficient	0.985	0.999	0.999	0.999

A fixed time of 20 minutes was established as the most suitable time (best r-value) to meet these requirements and to give more reproducible absorbances with low standard deviations.

The sequence of addition of the reagents as described under calibration graph was essential for good reproducibility. The formed color was found to remain stable for at least 24 hours. The optimised conditions were utilised to construct the calibration graph using authentic cefprozil.

Beer's law was obeyed for drug concentrations within 5–25 µg/ml. Spectral data for the reaction of ascorbic acid with cefprozil are presented in Table 2. The low values of the standard deviations of the slope and correlation coefficient values (not less 0.999) reflected the consistency of the prepared calibration graphs. The limit of detection was calculated using the formula:

$$\text{L.O.D}={\text{Y}}_{\text{B}}+{3\text{S}}_{\text{B}}$$

where Y_B_ is blank signal, 3S_B_ is three standard deviations of the blank.

**Table 2:** Spectral data of the reaction of cefprozil with ascorbic acid

λ_max_ (nm)	Linearity range (μg/ml)	Limit of quantification (μg/ml)	Limit of detection (μg/ml)	Intercept	Slope	Correlation coefficient	Molar absorbitivity (L mol^−1^ cm^−1^)

408	5–25	3.21	0.96	0.02835 ± 0.033	0.0168 ± 0.001995	0.999	7.2 × 10^3^

The limit of quantification is defined as the lower limit for precise quantitative measurement as opposed to qualitative detection. The value of Y_B_ + 10S_B_ was used for this calculation (14). The accuracy of the procedure and freedom of interference by the tablets excipients was confirmed by the results obtained for recovery testing of added amount of authentic cefprozil to tablets solution in the ratio of 1:1. The calculation of added recovery was done using an adopted formula:

$$\frac{{\text{A}}_{\text{T}}-{\text{A}}_{\text{Sm}}}{{\text{A}}_{\text{Std}}}\times 100$$

where A_T_ is total absorbance of mixture (tablet solution + authentic solution), A_Sm_ is absorbance of sample solution and A_Std_ is absorbance by authentic solution.

The results showed good recovery (99.7% ± 1.9% (RSD), n=2). The reproducibility for a concentration of 15 µg/ml was found to be 100% ± 0.4% (RSD), n=3. This reflected the precision of between-run replicates. The method was applied for the drug uniformity testing in cefprozil^®^ 500 mg tablets where good assay results (X̄ ± RDS (%), n) were obtained (99.89 ± 0.87%, n=3) (Table 3).

**Table 3:** Validation results of the Method (A) compared to the USP method

	Content% of cefprozil ± RSD%	at cal t(tab)	aF cal F(tab)

Method A	99.89 ± 0.87% (n=3)	1.63 (2.78)	4.33 (19)
Liquid chromatography method	98.0 ± 1.81% (n=3)		

a values for *t* and *F* calculated and tabulated.

Tablets solutions prepared simulating the calibration graph (5–25 µg/ml), further confirmed the good assay results. The plots were almost superimposed on each other. The slopes were used to calculate the tablets content using the following formula:

$$\frac{\text{Mean}\;\text{slope}\;\text{of}\;\text{sample}\;\text{graph}}{\text{Mean}\;\text{slope}\;\text{of}\;\text{authentic}}\times 100$$

This sample calibration graph can also be considered a useful indication of absence of excipients interference (superimposed parallel graphs) as it uses a range of low to high concentrations of sample solution (5–25 µg/ml). Bulk and sample calibration graphs obtained for method A were r=0.9971, slope=0.01794, intercept=0.0133 for standard and r=0.9977, slope=0.01793, intercept=0.0138 for sample (n=3). The average percent recovery was 100.69% ± 0.81 (n=3) for each concentration (5–25 µg/ml). The bathochromic shift exerted by the formed colored species may also eliminate any possible interference.

The validity of the method for the determination of cefprozil in bulk form was assessed by comparison of the statistical results obtained with those of the official USP liquid chromatography. Data of Table 3 show the obtained assay results and the calculated *t*- and *F*-values as compared to the corresponding tabulated values at 95% confidence level. As the calculated *t*- and *F*-values were less than tabulated ones, the result of this method can be considered as accurate and precise as the liquid chromatography method. It can be recommended for drug determination in quality control and routine analysis of the drug.

**Figure 2:**
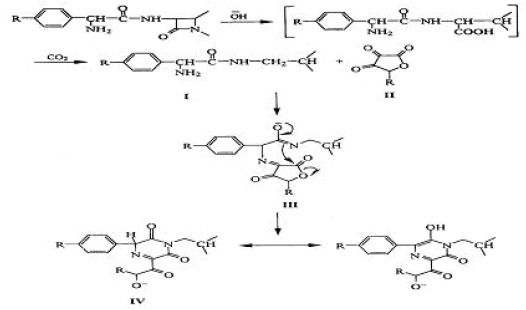
Proposed reaction pathway of cefprozil colour product.

According to the molar ratio method utilizing equimolar solutions of cefprozil and ascorbic acid (3 × 10^−3^M), the reaction stiochiometry was found to be 1:1 ratio. Accordingly the proposed reaction pathway between the drug and the reagent is expected to proceed as in Fig. 2:

Figure 2 is based on the pathway suggested for cephalosporins having α-aminoacyl function (13).

An important advantage of the method is the possible determination of the degraded form of the drug and hence the determination of the intact drug through the difference spectroscopic method (ΔA). The application of this stability-indicating procedure to cefprozil in bulk form or in tablets form indicated a purity of about 95% for cefprozil in both bulk and tablets dosage form. The degraded amount of cefprozil in bulk form was 4.93% ± 0.17 SD, n=3 and 5.5% ± 0.28 SD, n=3 for the dosage form.

### Method B

During the study of the optimum conditions for the reaction of cefprozil with ascorbic acid (Method A) the formation of a faint color was noticed when 0.1N NaOH was added to the solution of cefprozil. The intensity of the color was observed to increase with time at room temperature. This observation tempted the authors to study the direct reaction of cefprozil with NaOH at different concentrations (0.2, 0.5, and 1.0N), and to investigate the possible use of the reaction as a direct quantitative method.

**Figure 3:**
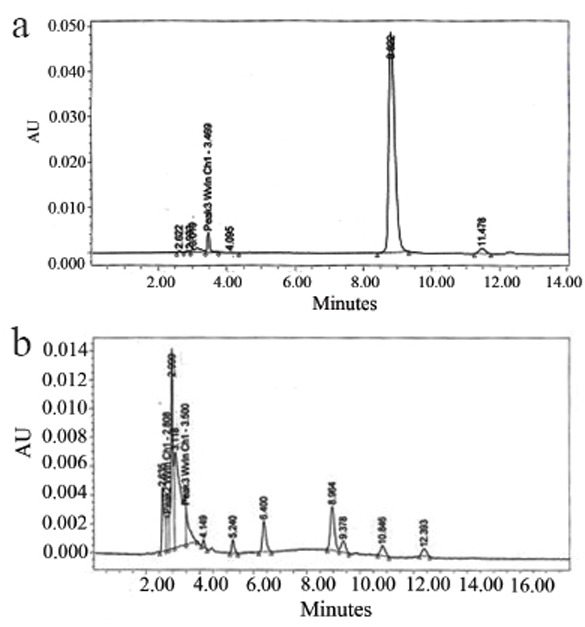
a, Liquid chromatography chromatogram of cefprozil standard in 1N NaOH (zero time); b, Liquid chromatography chromatogram of cefprozil standard in 1N NaOH (30 minutes).

**Study of the degradation of cefprozil with NaOH:** This study was carried utilizing the USP liquid chromatography method. Fig. 3a and Fig. 3b illustrate typical chromatograms of cefprozil at zero time and upon standing in 1N NaOH for 30 minutes at room temperature, respectively.

**Figure 4:**
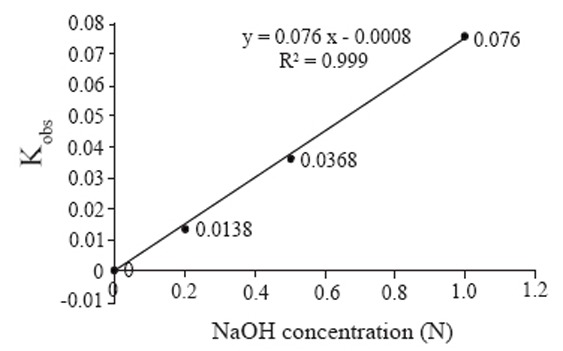
Effect of NaOH concentration on the degradation constant (K_obs_) of cefprozil at pH >12 at room temperature (25 ± 1).

The disappearance of the intact drug under the influence of sodium hydroxide at different concentrations and different times was used to calculate the degradation rate constant (K_obs_) using regression analysis data of log peak area vs. time t (min). The reaction was a first-order reaction. Table 4 shows the slopes of the plots of effect of the different normalities of NaOH on cefprozil, and the corresponding K_obs_ values. Fig. 4 illustrates the effect of sodium hydroxide on the degradation constant (K_obs_) at pH>12 and room temperature. Table 5 shows the calculated t½ and t_90_ values corresponding to the calculated K_obs_. The outcome of these experiments and calculations revealed an increase in color intensity with increased sodium hydroxide normality and subsequent increase in the rate of the reaction which affects t½ and t_90_. This study also pointed that 1N NaOH was suitable for the fast drug hydrolysis even at room temperature. However, heating at 100°C catalysed the reaction with resultant high color intensity, within short time (15 minutes). The established optimized conditions for this direct assay were as indicated under section [Calibration graphs (Method B)].

**Table 4:** Values of K_obs_ of the degradation curves with different NaOH concentrations

Concentration (N)	Slope	K_obs_

0.2	−0.006	0.0138
0.5	−0.016	0.0368
1	−0.033	0.076

**Table 5:** Values of t_1/2_ and t_90_ of cefprozil calculated from K_obs_

Concentration mol/L	K_obs_	t_1/2_ minutes	t_90_ minutes

0.2	0.0138	50.22	7.64
0.5	0.0368	18.83	2.86
1	0.076	9.12	1.38

The reaction product was found to remain stable for at least 3 hours.

**Tablets assay results:** The validity of the method for the determination of cefprozil in bulk form and tablet form was assessed by statistical comparison of the results with those obtained by the official USP HPLC method. Table 6 shows the data obtained for calibration graph, limit of detection, and limit of quantification. Bulk and sample calibration graphs obtained for method B were r=0.996, slope=0.02014, intercept=2.7 × 10^−3^ for standard and r=0.9995, slope=0.01996, intercept=6.3 × 10^−3^ for sample (n=3). The average percent recovery was 98.75% ± 0.61 (n=3) for each concentration (5–25 µg/ml).

**Table 6:** Spectral data for the reaction of NaOH with cefprozil

λ_max_ (nm)	Linearity range (μg/ml)	Limit of quantification (μg/ml)	Limit of detection (μg/ml)	Intercept	Slope	Correlation coefficient	Molar absorbitivity (L mol^−1^ cm^−1^)

486	5–25	3.089	0.93	0.003 ± 0.0378	0.02 ± 0.00114	0.999	7.4 × 10^3^

The results obtained were in good agreement with the labeled amount and confirmed the comparable accuracy and precision of this method with the official method (Table 7) and method A of the present study.

**Table 7:** Validation results of Method (B) compared to the USP method

	Content% of cefprozil ± RSD%	at cal, t(tab)	aF cal, F(tab)

Method B	98.90 ± 0.0.50% (n=3)	0.83 (2.78)	13.21 (19)
Liquid chromatography method	98.0 ± 1.81% (n=3)		

a *t* and *F* calculated and tabulated

It is worth noting that the application of the official USP liquid chromatography method revealed its stability-indicating property (Fig. 3a and Fig. 3b).

**Figure 5:**
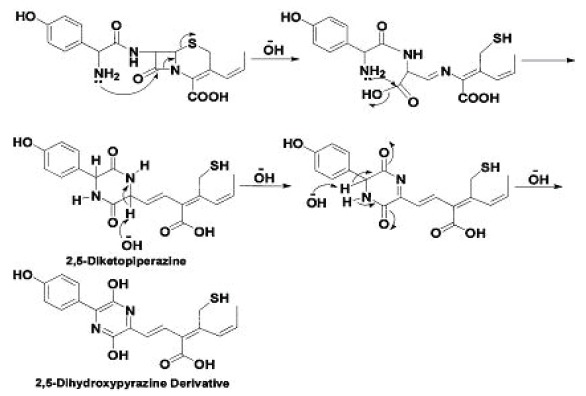
Proposed pathway for the reaction of cefprozil with NaOH.

**Proposed scheme for the alkaline hydrolysis of cefprozil:** Some studies (15) suggested that β-lactams with a free a-amino function (e.g. Cefaclor) form, under alkaline conditions, a cyclic 2,5-diketopiperazine derivatives absorbing at about 340 nm. The present study has shown that treating Cefprozil with 1N NaOH produced a colored species absorbing maximally at 486 nm. This is attributed to a base-catalyzed deprotonation and tautomerism of the 2,5-diketopiperazine to form a 2,5-dihydroxypyrazine derivative with an extended conjugated system (Fig. 5) that leads to the bathochromic shift observed.

## CONCLUSION

The above reported methods for the determination of cefprozil in the pure form and in pharmaceutical preparation, proved to be simple, precise, accurate and economic. All compounds containing β-lactam ring absorb in the range 250–270 nm which reflects non-specificity. The bathochromic shift obtained by reacting cefprozil with ascorbic acid is considered selective for cephalosporines with free α-aminoacyl group. In the present work, method B is considered more specific and selective for cefprozil in particular as it gives a product absorbing at 486 nm, a property not shared with other cephalosporines as these, under such conditions, absorb at about 325–340 nm. The suggested method can be used for the determination of cefprozil in quality control or industry.
